# “A Major Quality of Life Issue”: A Survey-Based Analysis of the Experiences of Adults With Laryngotracheal Stenosis with Mucus and Cough

**DOI:** 10.1177/00034894211050627

**Published:** 2021-10-08

**Authors:** Gemma M. Clunie, Catherine Anderson, Matthew Savage, Catherine Hughes, Justin W. G. Roe, Gurpreet Sandhu, Alison McGregor, Caroline M. Alexander

**Affiliations:** 1 Imperial College London, London, UK; 2 Imperial College Healthcare NHS Trust, London, UK; 3 Australian Market and Social Research Society, Glebe, NSW, Australia

**Keywords:** laryngotracheal stenosis, survey, mucus, cough

## Abstract

**Objectives:**

To investigate how the symptoms of mucus and cough impact adults living with laryngotracheal stenosis, and to use this information to guide future research and treatment plans.

**Methods:**

A survey was developed with the support of patient advisors and distributed to people suffering with laryngotracheal stenosis. The survey comprised 15 closed and open questions relating to mucus and cough and included the Leicester Cough Questionnaire (LCQ). Descriptive statistics, *X*^2^ and thematic analyses were completed.

**Results:**

In total, 641 participants completed the survey, with 83.62% (n = 536) reporting problems with mucus; 79% having daily issues of varying severity that led to difficulties with cough (46.18%) and breathing (20.90%). Mucus affected voice and swallowing to a lesser degree. Respondents described a range of triggers; they identified smoky air as the worst environmental trigger. Strategies to manage mucus varied widely with drinking water (72.26%), increasing liquid intake in general (49.35%) and avoiding or reducing dairy (45.32%) the most common approaches to control symptoms. The LCQ showed a median total score of 14 (interquartile range 11-17) indicative of cough negatively affecting quality of life. Thematic analysis of free text responses identified 4 key themes—the Mucus Cycle, Social impact, Psychological impact, and Physical impact.

**Conclusion:**

This study shows the relevance of research focusing on mucus and cough and its negative impact on quality of life, among adults with laryngotracheal stenosis. It demonstrates the inconsistent advice and management strategies provided by clinicians for this issue. Further research is required to identify clearer treatment options and pathways.

## Introduction

Acquired laryngotracheal stenosis (LTS) is a rare condition characterized by any narrowing of the upper airway involving the larynx and/or trachea. There are multiple causes of the condition in adults including intubation and tracheostomy, autoimmune conditions or idiopathic disease.^1^ The most common symptoms reported by patients include breathlessness and stridor.^2^ Patients often experience a gradual deterioration of respiratory symptoms between surgical procedures to manage the condition, with more definitive procedures such as laryngotracheal reconstruction aiming to resolve symptoms completely.^3^

Whilst dyspnea remains the most severe problem experienced by patients with acquired LTS, recent research has demonstrated that symptoms and functional impact of the condition are multifactorial and can encompass difficulties with voice and swallow.^4-6^ A recent qualitative study investigating the lived experience of LTS and reconstructive procedures explored the symptom impact of “furball moments,”^7^ a term used to describe thick, tenacious secretions that required conscious clearance prior to eating and drinking. The symptoms of mucus and cough were described in the context of swallowing but were clearly problematic to participants and their lives.

These difficulties have been acknowledged within literature relating to LTS but there remains a lack of research into mucus and cough.^8,9^ Clinical advice for patients complaining of difficulties managing mucus or experiencing cough focuses on the use of nebulizers with 1 survey reviewing nebulizer use for adults with subglottic stenosis (SGS).^8^ However, there is an absence of research concerning either symptom profile or treatment for these difficulties. In the context of the current COVID-19 pandemic, it is also likely that chronic difficulties with cough and mucus production may have negative implications for patients due to cough stigma.^10,11^

Mucus and cough are problematic features of other chronic health conditions such as chronic obstructive pulmonary disease (COPD)^12^ and head and neck cancer,^13^ whether as a result of diagnosis or treatment. These symptoms have significant quality of life implications, with a recent exploratory study of patients with COPD using an online patient community to demonstrate the impact that their mucus and cough symptoms caused in every aspect of life.^14^

As a preliminary step in learning more about mucus and cough in adults with LTS, and to gather information from a broad population to guide future research plans, an online survey was developed. The aim of the survey was to investigate the experience of living with mucus and cough symptoms in adults with LTS, to better understand how mucus and cough impact patient’s lives and guide clinical care.

## Methods

### Survey Design

With ethical approval (North East—Newcastle & North Tyneside 2 Research Ethics Committee (18/NE/0341)), a survey study was designed to investigate patient experiences of mucus and cough on a background of LTS. Questions were developed by the lead investigator and clinician (GC) using Qualtrics^XM^ software. The survey was then refined and updated by a patient-partner (CA). A patient advisory group and the multi-professional team then participated in face and construct validity, usability, and item reduction to maximize the relevance of the questions. The survey included 15 closed and open questions covering demographics, cause, and treatment of LTS and experiences and management of mucus. The patient experience of cough was evaluated using the 19-item patient-reported outcome measure, the Leicester Cough Questionnaire (LCQ)^15^ and a free text question. The full survey is available in Supplemental Appendix 1.

### Recruitment and Participants

An information sheet about the survey, including the Qualtrics^XM^ link was emailed to LTS patients a tertiary U.K center who had already participated in research or had agreed to be contacted about research. Inclusion criteria were a diagnosis of LTS and aged 18 years or over. The survey was also shared through social media including Twitter, an online support group (Living with Idiopathic Subglottic Stenosis; www.facebook.com/groups/idiopathicsubglotticstenosis) and an email database held by the patient-partner (CA). Although the support group title references a specific etiology of LTS it has members with a range of diagnosed causes.

A sample size calculation was completed based on a presumed population size of 3900 (total number of support group members at time of surveying and the original focus group participants) with a confidence level of 90% and a 10% margin of error. This estimated a sample of 67 responses.

The settings of the Qualtrics^XM^ software were adjusted to prevent duplication and to ensure that completion of the questionnaire was fully anonymous. The survey was open from 17 June 2020 to 4 July 2020, with 2 email reminders sent. Prior to completion participants were asked to read the information sheet with full details of the study and complete a consent form embedded into the survey as the first question.

### Data Analysis

The Qualtrics^XM^ online software was used to collect and collate the initial responses. Numerical data analysis was completed in Excel, with descriptive statistics used to analyze demographic details and responses from closed questions. A thematic analysis^16^ approach was used to analyze free text responses, with initial descriptive analysis (completed by CH) followed by interpretative analysis to establish broad meanings and experiences of participants. Analysis was completed by CH, MS, and GC, with verification by CA.

## Results

The survey was completed by 641 participants. This was a significant increase from the estimated sample size. Most respondents were female (97%) with 6 patients identifying as male (0.94%). Remaining participants either did not state biological sex or preferred not to say (1.72%). All participants were over 18, with the largest age group ranging between 45 and 54 (35.57%) and the eldest participant over 85. Idiopathic subglottic stenosis (iSGS) was the most common etiology (87.21%) with other etiologies including autoimmune disorders (2.96%), previous surgery (2.81%), and intubation/tracheostomy (2.18%). Most participants (92.67%) had had experience of surgery for their stenosis, ranging from dilatation and laser procedures to complex airway reconstruction. Full demographic details are provided in Table 1.

**Table 1.** Demographics of Survey Participants.

Demographic	Result n = 641
Age	% (n)
18-24	0.16 (1)
25-34	9.52 (61)
35-44	21.22 (136)
45-54	35.57 (228)
55-64	24.02 (154)
65-74	6.86 (44)
75-84	1.09 (7)
85+	0.16 (1)
No response	1.40 (9)
Sex	% (n)
Male	0.94 (6)
Female	97.35 (624)
Other/prefer not to say	1.72 (11)
Ethnicity	% (n)
White	95.00 (609)
Black	0.47 (3)
Asian	0.31 (2)
Mixed/multiple ethnic groups	1.40 (9)
Other	1.09 (7)
Prefer not to say	1.72 (11)
Cause of airway stenosis	% (n)
Autoimmune disease	2.96 (19)
Congenital	0.47 (3)
Idopathic subglottic stenosis	87.21 (559)
Intubation/prolonged tracheostomy	2.18 (14)
Surgery	2.81 (18)
Trauma	0.31 ()
Other	2.65 (17)
No response	1.40 (9)
Have you had surgery for stenosis?	% (n)
Yes	92.67 (594)
No	5.93 (38)
No response	1.40 (9)
Types of surgery reported	% (n)*
Crichotracheal resection	3.03 (32)
Endoscopic reconstruction	2.09 (22)
Laryngotracheal reconstruction	2.46 (26)
Tracheal resection	5.69(60)
Laser	34.22 (361)
Dilatation/stretch	47.96 (506)
Other	4.55 (48)

* Many participants had multiple surgeries so surgery is presented as a percentage of total number of surgeries (n = 1055).

**Figure 1.**
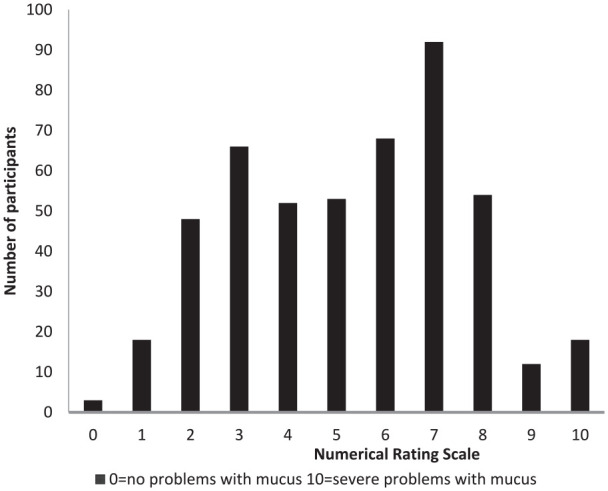
How would you rate your mucus problem?

The first symptom question was “Do you have any problems with mucus?” If participants selected no for this question, they were immediately taken to the LCQ. Problems with mucus were reported by 83.62% of all survey participants. Most experienced daily issues (79%), although a subjective rating of severity on a 0 to 10-point rating scale varied more broadly (see Figure 1) with more even distribution of responses.

**Figure 2.**
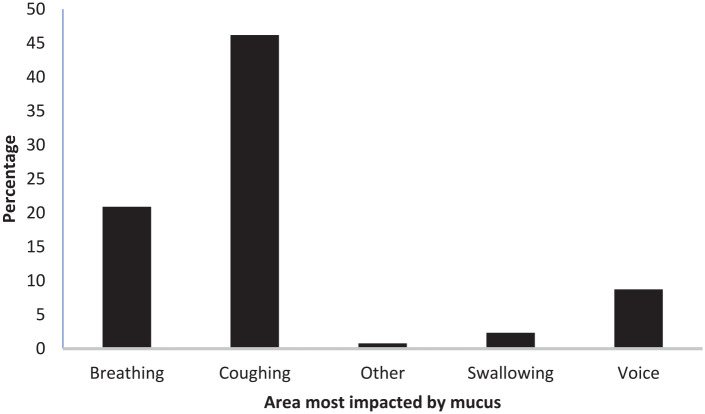
What area is most impacted by mucus?

Participants reported that mucus symptoms impacted coughing (46.18%), with others reporting that breathing (20.90%) and voice (8.74%) were most significantly affected by mucus (see Figure 2).

An open text response question explored when mucus started affecting participants. Due to the range of answers this was analyzed thematically with responses grouped into timing- or event-triggered. The most frequent responses were event-triggered: after surgery (18.41%) and before diagnosis (16.54%). Table 2 presents the full range of responses.

**Table 2.** When Did Mucus Start Affecting You?

Trigger	Demographic	Result % (n = 641)
Event	Before diagnosis	16.54 (106)
	After diagnosis	6.86 (44)
	Before surgery/when stenosis worsens	4.99 (32)
	After surgery	18.41 (118)
	Pregnancy	0.62 (4)
Timing	<1 year	0.47 (3)
	1-9 years	9.83 (63)
	10-20	5.62 (36)
	21+ years	1.87 (12)
	Always	6.24 (40)
Can’t remember		3.12 (20)
Chose not to respond		25.43 (163)

Table 3 shows responses to an impact rating scale for environmental triggers, with smoky air being the worst trigger, compared to clear air, humidity, cold and hot weather where there was no impact in either direction, *X*^2^ (20, n = 641) = 1104.64, *P* < .001.

**Table 3.** Frequency Distribution of Responses to What Impact do These Situations Have on Mucus.

Rating (0 = makes it worse 10 = makes it better)	Humidity	Smoky air	Clear air	Hot weather	Cold weather
0	52	125	21	47	47
1	34	73	1	38	31
2	33	***48***	2	33	38
3	44	36	3	42	49
4	29	26	8	32	***41***
5	***125***	75	177	***173***	161
6	22	4	***38***	32	31
7	41	5	49	19	19
8	35	4	58	14	15
9	17	4	37	8	16
10	28	8	92	13	11

*Note.* Mode: highlighted number. Median: bold, italicized number.

Participants were also given the opportunity to report other situations that had an impact on mucus and described a range of triggers, including exercise, stress, cigarette smoke, allergies or ill health, diet particularly dairy and alcohol, hydration, time of day and position, for example lying flat. Several participants reported that mucus worsened as they came closer to requiring treatment, and one wrote *“no matter the external environment, I have mucus problems all day, every day.”*

**Figure 3.**
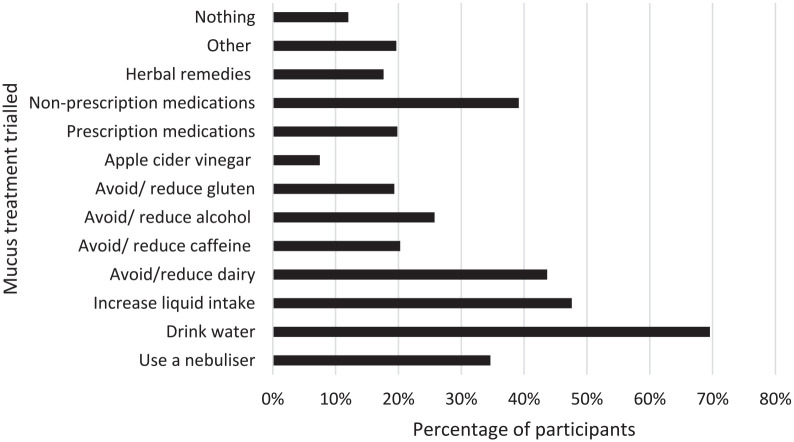
Types of treatments trialed to improve mucus.

The most frequently trialed treatments to manage mucus were drinking water (72.26%), increasing liquid intake in general (49.35%) and avoiding/reducing dairy (45.32%). Over a third of respondents had tried a nebulizer (36.29%) with a small proportion reporting that they had not attempted any treatments (12.90%). Figure 3 shows the full range of responses with many participants trying multiple options. Open text responses showed that participants had trialed a range of prescription and non-prescription medications such as proton-pump inhibitors (PPIs), antihistamines, antibiotics, steroids, nasal sprays, and expectorants. Herbal remedies were also trialed including apple cider vinegar, tea, essential oils, and turmeric.

The effectiveness of these treatments was rated by participants as completely, partially, or not at all effective. Table 4 shows the range of responses, with very few treatments in isolation reported as completely effective. A closer analysis of whether 1 specific medication type was more effective than others showed no consistency between category of medication and efficacy in relieving symptoms, although participants had often listed multiple medications in the free text response and had not differentiated which one was or was not effective. Of anecdotal interest was 1 participant who had been prescribed doxycycline for another condition (Lymes Disease) and consequently experienced a “90% reduction” in mucus.

**Table 4.** How Successful is Each Technique to Manage Mucus?

Technique	Completely	n	Partially	n	Not at all	n	Total
Use a nebulizer	14.98%	34	75.77%	172	9.25%	21	227
Drink water	6.28%	28	83.63%	373	10.09%	45	446
Increase liquid intake in general	5.94%	18	83.83%	254	10.23%	31	303
Avoid/reduce dairy	10.36%	29	80.71%	226	8.93%	25	280
Avoid/reduce caffeine	5.65%	7	70.97%	88	23.39%	29	124
Avoid/reduce alcohol	6.45%	10	68.39%	106	25.16%	39	155
Avoid/reduce gluten	8.00%	10	68.80%	86	23.20%	29	125
Take apple cider vinegar	2.04%	1	57.14%	28	40.82%	20	49
Take prescription medications	12.50%	11	63.64%	56	23.86%	21	88
Take non-prescription medications	8.33%	15	79.44%	143	12.22%	22	180
Take herbal remedies	5.17%	3	79.31%	46	15.52%	9	58
Other	23.81%	10	71.43%	30	4.76%	2	42
Nothing	15.38%	2	15.38%	2	69.23%	9	13

Treatments were usually recommended by doctors or the online support group (see Table 5) however other healthcare professionals or friends were also sources of advice.

**Table 5.** Who Recommended It?

Technique	Doctor	Pharmacist	Speech therapist	Nurse	Support group	Naturopath	Friend	Other	Total
Use a nebulizer	62.81%	0.00%	1.51%	1.51%	31.66%	1.01%	1.01%	0.50%	199
Drink water	32.34%	0.00%	2.17%	0.82%	37.77%	1.63%	3.80%	21.47%	368
Increase liquid intake in general	32.00%	0.00%	2.80%	2.00%	37.60%	2.00%	2.80%	20.80%	250
Avoid/reduce dairy	14.11%	0.00%	1.24%	0.83%	50.21%	6.64%	3.32%	23.65%	241
Avoid/reduce caffeine	15.79%	0.00%	3.51%	0.88%	39.47%	7.02%	5.26%	28.07%	114
Avoid/reduce alcohol	20.00%	0.00%	2.96%	0.00%	37.78%	5.93%	3.70%	29.63%	135
Avoid/reduce gluten	14.02%	0.93%	0.93%	1.87%	39.25%	14.95%	4.67%	23.36%	107
Take apple cider vinegar	7.14%	0.00%	0.00%	0.00%	40.48%	4.76%	4.76%	42.86%	42
Take prescription medications	91.78%	0.00%	0.00%	0.00%	4.11%	0.00%	0.00%	4.11%	73
Take non-prescription medications	50.94%	2.52%	0.63%	1.26%	24.53%	2.52%	3.77%	13.84%	159
Take herbal remedies	2.00%	0.00%	2.00%	0.00%	16.00%	26.00%	12.00%	42.00%	50
Nothing	50.00%	0.00%	0.00%	0.00%	0.00%	0.00%	0.00%	50.00%	6
Other	21.05%	0.00%	0.00%	0.00%	36.84%	2.63%	2.63%	36.84%	38

### Leicester Cough Questionnaire

**Figure 4.**
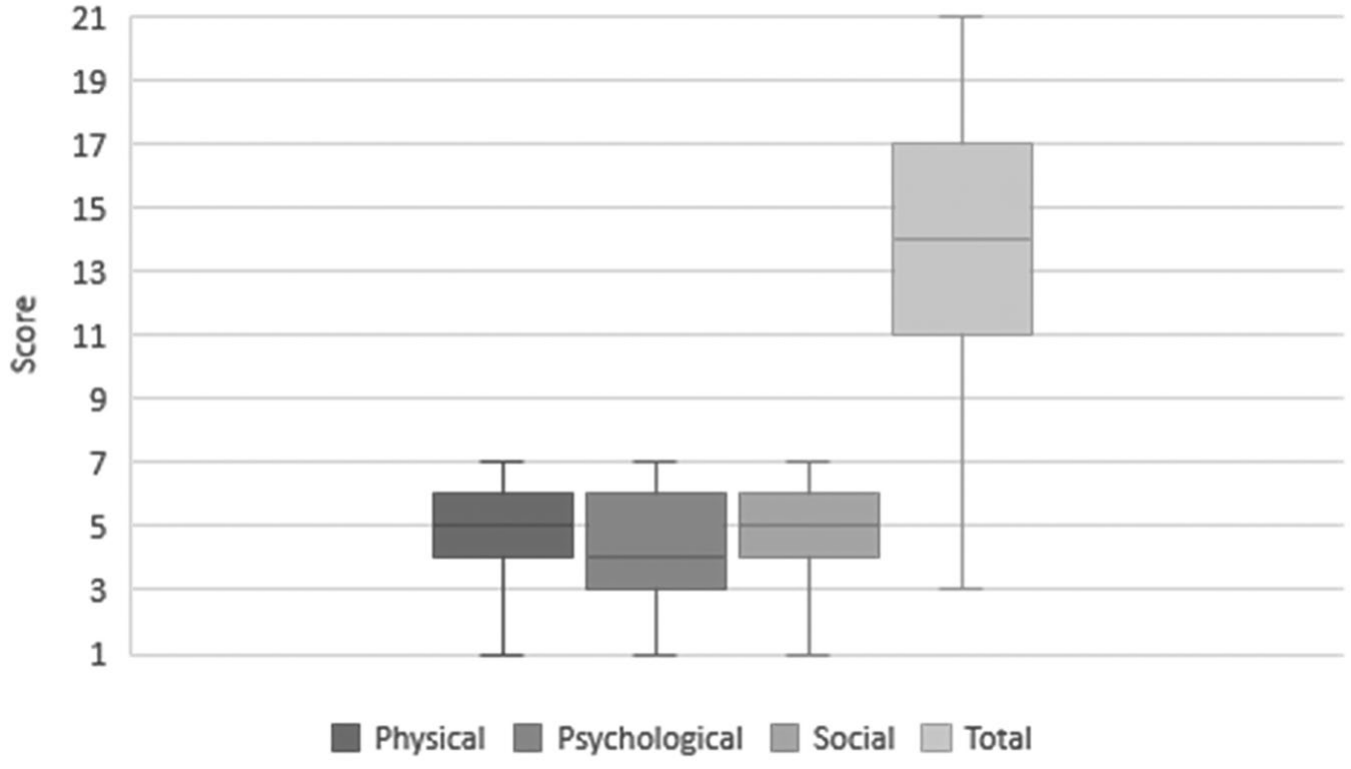
Box and whisper plot of median and interquartile range for Leicester cough questionnaire scores.

Analysis of the LCQ is presented in Figure 4. There are 3 separate domains: Physical, Psychological and Social, with each scored from 1 to 7. The total score is the sum of these domains and ranges from 3 to 21, with a higher score indicative of better quality of life. The median total score was 14 (interquartile range 11-17). Sub-analysis of the data to compare whether participants who reported no problems with mucus scored differently on the LCQ to those who did revealed no difference in score.

### Open Text Responses

Thematic analysis of open text responses for question 8 “Is there anything else you would like to say about the impact of mucus on your life?” and question 36 “Is there anything else you would like to say about the impact of cough on your life?” identified 4 overarching themes related to mucus and cough: (1) Mucus Cycle, (2) Social Impact, (3) Physical Impact, and (4) Psychological Impact. Each overall theme is discussed in detail below:

#### Mucus cycle

Participants reported a cycle of mucus and cough symptoms worsening in relation to the narrowness of their airway and the cycle of surgery. There was consistent reporting of a need to adapt to mucus and cough as part of life with the unpredictability causing issues for some:

*“It’s very frustrating - I can be absolutely fine, then it sticks to my vocal cords and I cannot talk”*

Most respondents described a pattern of mucus and cough worsening as their stenosis did, with some using it as a measure of where in the treatment cycle they were:

*“The closer I get to needing surgery, the worse it gets. My husband uses it as a monitor to see when he needs to suggest that I go get scoped (after 45 surgeries, it’s sad that we know the signs. . . that well).”*

A few respondents reported that their mucus problems had resolved post-surgery but there were a small number of participants who felt that mucus and cough worsened following surgery:

*“The days during week 2 and 3 after laser/dilation are always problematic.”*

The cycle also referred to variability in mucus and cough, related to time during the day or night, activities, allergies and the weather or season. Participants reported finding that they must adapt their lives to manage mucus according to the point in the cycle they had reached:

*“My cough definitely impacts my sleep and is very frustrating. On the days following a tough night I am not as productive during the day.”*

Part of the cycle also related to how to manage symptoms. Different techniques and treatments were recommended to help, with respondents describing a fine balance to best manage these. For others there was ongoing challenge and frustration that they received little guidance or understanding from their healthcare professionals:

*“I’ve been told by doctors I’m a mucus producing machine and no doctor has anything more to suggest.”*

*“Folks may say use a nebulizer, but nobody is recommending HOW MUCH per day.”*

There was a clear sense that mucus and cough were a significant aspect of living with LTS with wide-reaching impact.

#### Social impact

One of the main impacts expressed by participants was the effect of mucus and cough on their social connections and working lives. There was a sense of isolation and loss of social interactions as a direct result of their symptoms:

*“It is terrible at work - I talk all day as a physician and often have to step out of the room to go clear mucus because it collects at my vocal cords and stops my speech suddenly. I also do not want a relationship any longer as the coughing episodes are pretty gross, so I choose to remain single in part because of the mucus and cough.”*

The sense of isolation was also compounded by embarrassment or discomfort that frequent coughing bouts, or the need for mucus clearance, would mis-identify them to others as acutely unwell. This led to social distress, and either the need to *“explain to everyone that [the cough relates to] a chronic airway issue,”* or further avoidance of social interactions. Participants also reported that friends, family, and colleagues experienced *“unnecessary concern”* due to them noticing the constancy of symptoms.

The COVID-19 pandemic was referred to repeatedly as a significant negative factor related to social activities due to the stigma attached to coughing and mucus.

*“My cough embarrasses me in meetings and when I am teaching all the time. I hate having to cough loudly and persistently. I avoid public places. . .in case people think I have COVID-19.”*

A few responses also indicated that the necessary public health precautions for COVID-19 were challenging and impinged on social interactions and daily life:

*“With COVID-19, I hesitate to go into stores due to the mask increasing the phlegm.”*

#### Physical impact

Another key theme related to the physical effects of mucus and cough, whether this related to changes in voice, impact on sleep, or ability to exercise.

*“I am also avoiding exercise as it is always much worse when I exercise. This is impacting my fitness, weight and mental health”*

Participants reported pain caused by repeated coughing, as well as choking, laryngeal discomfort, and constant irritation. Of significant concern to some respondents was the potential for coughing or mucus to exacerbate their underlying condition:

*“I am concerned mucus and harder coughing increases the rate of stenosis growth via the constant irritation”*

A small minority of respondents drew attention the negative impact of coughing on pelvic floor strength leading to incontinence issues. The physical impact of mucus and cough was extrapolated as an example of how challenging living with LTS was, and how detrimental to quality of life:

*“This whole condition needs more attention and care in every respect. Subglottic stenosis is a horrible disease to have. Before my resection, no sane person could ever tolerate sleeping in the same room with me.”*

#### Psychological impact

The final theme related to the psychological impact mucus and cough had on respondents. Whilst a small minority of participants did not feel their symptoms impacted them negatively or at all, for the majority their experiences were “*annoying at best, frightening at worst.”* A repeated concern was fear of dying in your sleep due to a mucus plug, and for many their worries about the future were intimately related to management of their mucus and cough. One participant reported:

*“This is a major quality of life issue for us. Plugs have killed and there does not seem to have been any attention or research into prevention of this side effect. Our quality of life would increase greatly if this issue was addressed.”*

Mood was negatively impacted by mucus and cough, with anxiety and depression linked with symptoms. Control of symptoms was also key to managing the impact on quality of life with participants expressing that *“When [mucus and cough are] not under control it makes life even more challenging.”* This was then exacerbated by how little is known about how to best manage the symptoms and trial and error nature of their management.

## Discussion

The aim of the survey was to investigate whether people living with LTS experience mucus and cough symptoms due to their condition, and how this impacts their lives with a view to guiding clinical care and further research.

The response rate to the survey exceeded expectations and demonstrated the importance of the subject matter to people living with LTS. Most participants (83.62%; n = 536) reported difficulties with mucus, with nearly half (46.18%; n = 248) reporting that the biggest impact on their lives related to coughing. This correlated with the median score of the LCQ (14; ICQ 11-17) indicating reduced quality of life. The link between mucus and cough is well established in respiratory conditions such as asthma and COPD.^17^ Responses to this survey indicate that there is a need to establish a clearer understanding of the symptoms in relation to LTS, rather than just their relevance to differential diagnosis.^18^

One of the key findings of the survey was inconsistent advice relating to management of treatment of mucus and cough symptoms. A previous survey of 316 participants^8^ reported that nearly half (45.57%: n = 144) of their survey respondents had used nebulizers to manage mucus for subglottic stenosis (SGS) with no standardized solution used. In our survey a third (36.26%, n = 232) of participants reported using a nebulizer, often without clear guidelines about how often to nebulize, or what solution to use. The lack of clear treatment protocols for managing mucus and cough was apparent in all survey responses. Further research is required to develop consistent management practices similar to those that exist for asthma^19^ and COPD^20^ thereby benefiting people with LTS.

Mucus and cough symptoms were particularly problematic in the context of COVID-19 leading to reports of increased anxiety and stress consistent with other research relating to LTS.^21^ However, many respondents reported that their difficulties predated the pandemic, demonstrating that mucus and cough require greater attention by clinicians to manage the distress and impaired quality of life.

The survey design process was aimed to minimize question ambiguity, but this may still have influenced responses. A survey study is always limited by design as there is no way to control for recall bias or the demographic makeup of the respondents. Most respondents were female, with a high proportion of idiopathic SGS due to the membership of the Facebook group. Future research relating to this area needs to determine if men and people with differing etiologies for their stenosis experience similar issues. Another limitation of the survey design was that it did not allow for detailed sub-analysis of treatment trialed, for example pharmaceutical category compared with treatment efficacy. Future research to determine the efficacy of specific mucus treatments would be beneficial to both patients and clinicians. Despite this, our findings show that mucus and cough are of significant concern to adults with LTS and therefore of interest to researchers.

## Conclusions

This survey is a first analysis of the impact of mucus and cough on adults with LTS. Most respondents experienced difficulties with mucus and cough. They reported their frustrations that the symptoms were not given more consideration by their clinicians given its negative impact on quality of life. Further research is needed to develop evidence-based treatment options and better understanding of how and why these symptoms impact adults with LTS.

## Supplemental Material

sj-pdf-1-aor-10.1177_00034894211050627 – Supplemental material for “A Major Quality of Life Issue”: A Survey-Based Analysis of the Experiences of Adults With Laryngotracheal Stenosis with Mucus and Cough Click here for additional data file. Supplemental material, sj-pdf-1-aor-10.1177_00034894211050627 for “A Major Quality of Life Issue”: A Survey-Based Analysis of the Experiences of Adults With Laryngotracheal Stenosis with Mucus and Cough by Gemma M. Clunie, Catherine Anderson, Matthew Savage, Catherine Hughes, Justin W. G. Roe, Gurpreet Sandhu, Alison McGregor and Caroline M. Alexander in Annals of Otology, Rhinology & Laryngology

## References

[1] Gelbard A Francis DO Sandulache VC Simmons JC Donovan DT Ongkasuwan J. Causes and consequences of adult laryngotracheal stenosis. Laryngoscope. 2015;125(5):1137-1143. 25290987 10.1002/lary.24956 PMC4562418

[2] Almanzar A Danckers M. Laryngotracheal Stenosis. StatPearls Publishing; 2021:https://www.ncbi.nlm.nih.gov/books/NBK554561/ Available from. 32119448

[3] Ashiku SK Kuzucu A Grillo HC , et al. Idiopathic laryngotracheal stenosis: effective definitive treatment with laryngotracheal resection. J Thorac Cardiovasc Surg. 2004;127(1):99-107. 14752419 10.1016/j.jtcvs.2002.11.001

[4] Fiz I Koelmel JC Piazza C , et al. Predictors of recurrence after surgical treatment of idiopathic progressive subglottic stenosis. Acta Otorhinolaryngol Ital. 2018;38(5):417-423. 30498269 10.14639/0392-100X-1872 PMC6265673

[5] Hentze M Schytte S Pilegaard H Klug TE. Single-stage tracheal and cricotracheal segmental resection with end-to-end anastomosis: outcome, complications, and risk factors. Auris Nasus Larynx. 2019;46(1):122-128. 29934237 10.1016/j.anl.2018.06.001

[6] Clunie GM Roe JWG Alexander C Sandhu G McGregor A. Voice and swallowing outcomes following airway reconstruction in adults: a systematic review. Laryngoscope. 2021;131:146-157. 31943240 10.1002/lary.28494 PMC7754401

[7] Clunie GM Belsi A Roe JWG Alexander CM Sandhu G McGregor A. Not just dyspnoea: swallowing as a concern for adults with laryngotracheal stenosis undergoing airway reconstruction. Dysphagia. Online ahead of print. 2021. 10.1007/s00455-021-10287-3 PMC8948149 33830348

[8] Tanner K Anderson C Smith ME. Nebulizer use in adults with subglottic stenosis: a survey study. Ann Ushn Nos Laryngol. 2019;128(4):345-351. 10.1177/0003489418823797 30638026

[9] Reza Nouraei SA . Intubation-Related Tracheal Stenosis. In: Allen J Sandhu GS Nouraei SA , eds. Laryngology: A Case-Based Approach. 1st ed. London: Plural Publishing; 2019:399-445.

[10] Li Y Twersky S Ignace K , et al. Constructing and communicating COVID-19 stigma on twitter: a content analysis of tweets during the early stage of the COVID-19 outbreak. Int J Environ Res Public Health. 2020;17(18):6847. 10.3390/ijerph17186847 PMC7557581 32961702

[11] Baldassarre A Giorgi G Alessio F Lulli LG Arcangeli G Mucci N. Stigma and discrimination (SAD) at the time of the SARS-CoV-2 pandemic. Int J Environ Res Public Health. 2020;17(17):6341. 10.3390/ijerph17176341 PMC7503800 32878180

[12] Tian P-W Wen F-Q. Clinical significance of airway mucus hypersecretion in chronic obstructive pulmonary disease. J Transl Int Med. 2015;3(3):89-92. 27847895 10.1515/jtim-2015-0013 PMC4936458

[13] Pezdirec M Strojan P Boltezar IH. Swallowing disorders after treatment for head and neck cancer. Radiol Oncol. 2019;53(2):225-230. 31194691 10.2478/raon-2019-0028 PMC6572490

[14] Cook N Gey J Oezel B , et al. Impact of cough and mucus on COPD patients: primary insights from an exploratory study with an online patient community. Int J Chron Obstruct Pulmon Dis. 2019;14:1365-1376. 31417250 10.2147/COPD.S202580 PMC6599966

[15] Birring SS Prudon B Carr AJ Singh SJ Morgan MD Pavord ID. Development of a symptom specific health status measure for patients with chronic cough: Leicester Cough questionnaire (LCQ). Thorax. 2003;58(4):339-343. 12668799 10.1136/thorax.58.4.339 PMC1746649

[16] Braun V Clarke V. Using thematic analysis in psychology. Qual Res Psychol. 2006;3(2):77-101.

[17] Fahy JV Dickey BF. Airway mucus function and dysfunction. New Engl J Med. 2010;363(23):2233-2247. 21121836 10.1056/NEJMra0910061 PMC4048736

[18] Kheir F Rivera E Majid A. An adolescent with dyspnea and cough. A case of congenital tracheal stenosis. Am J Respir Crit Care Med. 2017;196(7):e30-e31. 10.1164/rccm.201704-0682IM 28683205

[19] BTS/SIGN. British Guideline on the Management of Asthma. 2019.

[20] NICE. Chronic Obstructive Pulmonary Disease in Over 16s: Diagnosis and Management [NG115]. In: NICE, 2019. 31211541

[21] Anderson C Sandhu G Al Yaghchi C. Impact of the COVID-19 pandemic on patients with idiopathic subglottic stenosis. Ear Nose Throat J. 2021;100(2_suppl):122s-130s. 10.1177/0145561320977467 33302743

