# Supplementary material for: “A Major Quality of Life Issue”: A Survey-Based Analysis of the Experiences of Adults With Laryngotracheal Stenosis with Mucus and Cough
Source: Ann Otol Rhinol Laryngol. 2021 Oct 8;131(9):962–70. doi: 10.1177/00034894211050627 (PMC9340141; doi:10.1177/00034894211050627)
Supplement: sj-pdf-1-aor-10.1177_00034894211050627 – Supplemental material for “A Major Quality of Life Issue”: A Survey-Based Analysis of the Experiences of Adults With Laryngotracheal Stenosis with Mucus and Cough [file sj-pdf-1-aor-10.1177_00034894211050627.pdf]

Airway stenosis patients, mucus and cough

Start of Block: Consent

- [ ] 1. I confirm that I have read and understand the subject information sheet dated 6 May 2020 version 1 part 2 for the above study and have had the opportunity to ask questions which have been answered fully.

- [ ] 2. I understand that my participation is voluntary, and I am free to withdraw at any time, without giving any reason and without my legal rights being affected.

- [ ] 3. I understand that my data will be automatically anonymised and I consent for the anonymised information collected to be used to support other research in the future, including those outside of the European Economic Area (EEA).

- [ ] 4. I give consent for my anonymised data to be used in future ethically approved studies. I give permission for my anonymised data to be sent to other organisations, including these outside of the EEA.

- [ ] 5. I consent to take part in the above study.

End of Block: Consent

Start of Block: Mucus questions

Q1 How old are you?

- ○ 18 - 24
- ○ 25 - 34
- ○ 35 - 44
- ○ 45 - 54
- ○ 55 - 64
- ○ 65 - 74
- ○ 75 - 84
- ○ 85 or older

Q13 What sex are you?

- ○ Male
- ○ Female
- ○ Other
- ○ Prefer not to say

Q12 What is your ethnic group? Please select one or more that best describes your ethnic background.

- ☐ White
- ☐ Black
- ☐ Asian
- ☐ Arab
- ☐ Mixed/Multiple ethnic groups
- ☐ Other, please describe: ____
- ☐ Prefer not to say

Q2 What is the reason for your airway stenosis?

- ○ Intubation/ Prolonged tracheostomy
- ○ Idiopathic Subglottic Stenosis
- ○ Autoimmune disease e.g. GPA, sarcoidosis, vasculitis
- ○ Trauma
- ○ Previous surgery, if so what? ____
- ○ Other ____
- ○ Congenital/ From childhood

Q14 Have you had surgery to manage your airway stenosis?

- ◯ Yes
- ◯ No

Q3 If you have had surgery, what surgery did you have? Select all that apply:

- ☐ Laryngotracheal reconstruction
- ☐ Endoscopic reconstruction (Maddern procedure)
- ☐ Tracheal resection
- ☐ Cricotracheal resection
- ☐ Laser
- ☐ Stretch/ Dilatation
- ☐ Other ____

Q4 Do you have any problems with mucus?

- ◯ Yes
- ◯ No

Q5 Over the last week, how bad would you rate your mucus problem on a scale of 0 to 10? (Where 0 means you had no problems with mucus and 10 means you have very bad problems with mucus)

	0 1 2 3 4 6 7 8 9 10
How do you rate your mucus?	[slider]

Q6 How often does mucus affect you?

- ○ Daily
- ○ Weekly
- ○ Monthly
- ○ More than monthly
- ○ Never

Q7 Select the ONE area your mucus impacts the most. Tell us more about how it impacts you:

- ○ Voice ____
- ○ Breathing ____
- ○ Coughing ____
- ○ Swallowing ____
- ○ Other

Q16 When did mucus start affecting you?

____

Q15 What impact do these situations have on mucus? Please move the slider along the line to indicate the impact level.

	Make it worse 0	No impact 5	Make it better 10
Humidity			
Smoky air			
Clear air			
Hot weather			
Cold weather			

Q9 What have you tried to help manage mucus?

- ☐ Use a nebuliser
- ☐ Drink water
- ☐ Increase liquid intake in general
- ☐ Avoid/ reduce dairy
- ☐ Avoid/ reduce caffeine
- ☐ Avoid/reduce alcohol
- ☐ Avoid/ reduce gluten
- ☐ Take apple cider vinegar
- ☐ Take prescription medications, please describe ____
- ☐ Take non-prescription medications, please describe ____
- ☐ Take herbal remedies, please describe ____
- ☐ Nothing
- ☐ Other ____

	How successful to manage mucus	Who recommended it?

	Completely	Partially	Not at all	Doctor	Pharmacist	Speech Therapist	Nurse	Support Group	Naturopath	Friend	Other
Use a nebuliser	◯	◯		◯	◯	◯	◯	◯	◯	◯	◯
Drink water	◯	◯		◯	◯	◯	◯	◯	◯	◯	◯
Increase liquid intake in general	◯	◯		◯	◯	◯	◯	◯	◯	◯	◯
Avoid/ reduce dairy	◯	◯		◯	◯	◯	◯	◯	◯	◯	◯
Avoid/ reduce caffeine	◯	◯		◯	◯	◯	◯	◯	◯	◯	◯
Avoid/ reduce alcohol	◯	◯		◯	◯	◯	◯	◯	◯	◯	◯
Avoid/ reduce gluten	◯	◯		◯	◯	◯	◯	◯	◯	◯	◯
Take apple cider vinegar	◯	◯		◯	◯	◯	◯	◯	◯	◯	◯
Take prescription medications, please describe	◯	◯		◯	◯	◯	◯	◯	◯	◯	◯
Take non-prescription medications, please describe	◯	◯		◯	◯	◯	◯	◯	◯	◯	◯
Take herbal remedies, please describe	◯	◯		◯	◯	◯	◯	◯	◯	◯	◯
Nothing	◯	◯		◯	◯	◯	◯	◯	◯	◯	◯
Other	◯	◯		◯	◯	◯	◯	◯	◯	◯	◯

Q10 Of these techniques to manage mucus, how successful is it, and who recommended it?

Q8 Is there anything else you would like to say about the impact of mucus on your life?

____

End of Block: Mucus questions

Start of Block: Leicester Cough Questionnaire. © 2001

Q17 These questions are designed to assess the impact of cough on various aspects of your life. Read each question carefully and answer by selecting the response that best applies to you. In the last 2 weeks:

	All the time	Most of the time	A good bit of the time	Some of the time	A little of the time	Hardly any of the time	None of the time
Have you had chest or stomach pains as a result of your cough?	○	○	○	○	○	○	○

Q18 In the last 2 weeks:

	Everytime	Most times	Several times	Some times	Occasionally	Rarely	Never
Have you been bothered by sputum (phlegm) production when you cough?	○	○	○	○	○	○	○

Q19 In the last 2 weeks:

	All of the time	Most of the time	A good bit of the time	Some of the time	A little of the time	Hardly any of the time	None of the time
Have you been tired because of your cough?	○	○	○	○	○	○	○

Q20 In the last two weeks:

	None of the time	Hardly any of the time	A little of the time	Some of the time	A good bit of the time	Most of the time	All of the time
Have you felt in control of your cough?	○	○	○	○	○	○	○

Q21 In the last two weeks:

	All of the time	Most of the time	A good bit of the time	Some of the time	A little of the time	Hardly any of the time	None of the time
How often have you felt embarrassed by your coughing?	○	○	○	○	○	○	○

Q22 In the last two weeks:

	All of the time	Most of the time	A good bit of the time	Some of the time	A little of the time	Hardly any of the time	None of the time
My cough has made me feel anxious	○	○	○	○	○	○	○

Q23 In the last two weeks:

	All of the time	Most of the time	A good bit of the time	Some of the time	A little of the time	Hardly any of the time	None of the time
Has interfered with my job, or other daily tasks	○	○	○	○	○	○	○

Q24 In the last two weeks:

	All of the time	Most of the time	A good bit of the time	Some of the time	A little of the time	Hardly any of the time	None of the time
I felt that my cough interfered with the overall enjoyment of my life	○	○	○	○	○	○	○

Q25 In the last two weeks:

	All of the time	Most of the time	A good bit of the time	Some of the time	A little of the time	Hardly any of the time	None of the time
Exposure to paints or fumes has made me cough	○	○	○	○	○	○	○

Q26 In the last two weeks:

	All of the time	Most of the time	A good bit of the time	Some of the time	A little of the time	Hardly any of the time	None of the time
Has your cough disturbed your sleep?	○	○	○	○	○	○	○

Q27 In the last two weeks:

	All of the time (continuously)	Most of the time	A good bit of the time	Some of the time	A little of the time	Hardly any of the time	None of the time
How many times a day have you had coughing bouts?	○	○	○	○	○	○	○

Q28 In the last two weeks:

	All of the time	Most of the time	A good bit of the time	Some of the time	A little of the time	Hardly any of the time	None of the time
My cough has made me feel frustrated	○	○	○	○	○	○	○

Q29 In the last two weeks:

	All of the time	Most of the time	A good bit of the time	Some of the time	A little of the time	Hardly any of the time	None of the time
My cough has made me feel fed up	○	○	○	○	○	○	○

Q30 In the last two weeks:

	All of the time	Most of the time	A good bit of the time	Some of the time	A little of the time	Hardly any of the time	None of the time
Have you suffered from a hoarse voice as a result of your cough?	○	○	○	○	○	○	○

Q31 In the last two weeks:

	None of the time	Hardly any of the time	A little of the time	Some of the time	A good bit of the time	Most of the time	All of the time
Have you had a lot of energy?	○	○	○	○	○	○	○

Q32 In the last two weeks:

	All of the time	Most of the time	A good bit of the time	Some of the time	A little of the time	Hardly any of the time	None of the time
Have you worried that your cough may indicate serious illness?	○	○	○	○	○	○	○

Q33 In the last two weeks:

	All of the time	Most of the time	A good bit of the time	Some of the time	A little of the time	Hardly any of the time	None of the time
Have you been concerned that other people think something is wrong with you, because of your cough?	○	○	○	○	○	○	○

Q34 In the last two weeks:

	Every time	Most times	A good bit of the time	Some of the time	A little of the time	Hardly any of the time	None of the time
My cough has interrupted conversation or telephone calls	○	○	○	○	○	○	○

Q35 In the last two weeks:

	Every time I cough	Most times when I cough	Several times when I cough	Some times when I cough	Occasionally when I cough	Rarely	Never
I feel that my cough has annoyed my partner, family or friends	○	○	○	○	○	○	○

Q36 Is there anything else you would like to say about the impact of cough on your life?

____

End of Block: Leicester Cough Questionnaire. © 2001
